# Deciphering the profiles of grapevine microbiomes from rhizosphere-to-leaf compartments using multi-omic analysis

**DOI:** 10.3389/fpls.2025.1734057

**Published:** 2026-01-26

**Authors:** Bo Wang, Zhuangwei Wang, Zhenxiao Chen, Jing Zhang, Xicheng Wang

**Affiliations:** 1 Institute of Pomology, Jiangsu Academy of Agricultural Sciences/Jiangsu Key Laboratory for Horticultural Crop Genetic Improvement, Nanjing, China; 2 College of Horticulture and Landscape Architecture, Tianjin Agricultural University, Tianjin, China

**Keywords:** bacteria, fungi, Muscadine, niche-shared microbes, stilbene compounds

## Abstract

**Introduction:**

Root- and leaf-associated microbiomes are crucial for plant health and influence the yield and quality of the products. The composition of microbes and their association with the host depend on different factors that must be continuously investigated.

**Results:**

We examined the composition and structure of bacterial and fungal communities in four compartments (root, rhizoplane, rhizosphere, and leaf endosphere) of two grapevine varieties (‘Alachua’ and ‘Noble’) targeting the 16S rRNA V5–V7 and ITS regions.

**Results and discussion:**

A comparison of the effects of the varieties and compartments showed that they were the major factors contributing to variations in the microbial structures. Bacterial alpha diversity significantly decreased from the rhizosphere to leaf endosphere, while the fungal alpha diversity did not show linear variations. According to normalized stochastic ratio analysis, deterministic processes dominated the bacterial and fungal assemblies of the leaf endosphere while stochastic processes in the rhizosphere and rhizoplane dominated the microbial assemblies. Assembly processes in bacterial and fungal roots differed (stochastic processes in bacteria and deterministic processes in fungi). Twenty shared core operational taxonomic units (OTUs) (bacteria, 13; fungi, 7) were identified across all compartments. Various stilbene compounds in leaf were significantly correlated with these shared core microbes, and weighted gene co-expression network analysis revealed that some hub genes were correlated with these metabolites. Thus, their role as regulators of grapevine microbiome interactions needs to be further evaluated. This study provided new profiles of the microbiota in different grapevines compartments, which suggested their association with grape metabolites and plant genes, representing a major development for further studies focused in understanding the role of these microorganisms for grapevine production.

## 1 Introduction

Diverse microorganisms are associated with various plant parts, from roots and stems to leaves, flowers, and fruits. These plant-associated microorganisms play crucial roles in host physiology, contributing to disease resistance, fitness, nutrient acquisition, and flavor chemistry (Petrushin et al., 2023; Walsh et al., 2024; Dubey and Sharma, 2021; Zhu et al., 2023; Zhang et al., 2022; Bai et al., 2022). Among various niches, the rhizosphere microbiome has received the most attention (Liu et al., 2022). Several studies have revealed a strong association between aboveground performance and root-associated microbiomes. For example, Yuan et al. (2018) found that root exudates recruit rhizosphere communities in response to aboveground pathogen infections. Pineda et al. (2020) showed that aboveground insect pests can be suppressed by conditioning the soil microbiome. However, few studies have focused on the specific relationships between leaf- and root-associated microbiomes.

The core microbiome constitutes the set of microbial taxa that are consistently found across individuals or within specific niches of a broader host population (Toju et al., 2018; Qiu et al., 2022). Zhang et al. (2022) reported that several core bacterial strains identified in maize stem xylem are nitrogen fixers. Different plant compartments with unique niches exhibit specific microbial associations (Chialva et al., 2022). Chen et al. (2020b) found that bacterial and fungal compositions vary among the rhizosphere, root endosphere, and stems of *Broussonetia papyrifera*. However, core microbiota studies have primarily investigated one organ. Thus, the core microbiota across different organs, particularly the aboveground and underground compartments, are poorly understood.

The assembly of compartment-specific microbiomes is governed by both environmental filters and host factors (Dastogeer et al., 2020; Friesen et al., 2011). However, compartment-specific microbial community structure and assembly are largely shaped by diverse host factors (Jones et al., 2019). Plants have a genetic basis for simultaneous interactions with many microbes (Beilsmith et al., 2019), and many host-driven microbiome assemblies have been reported. Tan et al. (2022) reported that tea genes related to the cell wall and carbon catabolism were strongly associated with the composition of the root endosphere microbial community. Liu et al. (2023b) reviewed the association between plant functional genes and rhizosphere microbes and claimed that the host genes recruit the plant microbiome by regulating crucial plant metabolites. Plant metabolites are vital signals or mediators of host-microbiome interactions. Research has shown that several metabolites, such as phenolic acids, organic acids, coumarins, benzoxazinoids, and terpenes, are associated with the rhizosphere microbiome (Pang et al., 2021; Wu et al., 2015; Harbort et al., 2020). However, the understanding of plant-metabolite-microbiome interactions is largely limited to the underground system, while the interactions with leaf metabolites, host genes, or leaf-associated microbiomes remain largely unknown.

Grapevines are significant perennial plants with high economic value. Studies have reported microbial variations in different grapevine compartments, including the roots, rhizosphere, leaves, flowers, cordons, canes, and sap (Bettenfeld et al., 2022; Zahid et al., 2021; Swift et al., 2023). Muscadine grapes (*Vitis rotundifolia* Michx.) are particularly notable for their high accumulation of stilbenes, a class of phytoalexins central to disease resistance (Louime et al., 2011; Balasubramani et al., 2019). However, few studies have investigated the core microbiota across the aboveground and underground compartments. Thus, the interaction between host-metabolite-microbiome in grapevines remains unclear.

In this study, the root, rhizoplane, rhizosphere, and leaves from two grapevine varieties were sampled during the berry ripening periods. Our aims were to (1) compare the microbial community compositions and structures across different compartments (the root endosphere, rhizoplane, rhizosphere, and leaf endosphere); (2) identify the shared core microbiota across these four compartments; and (3) elucidate the association among shared core microbes, stilbene compounds, and host genes at the transcriptome level.

## 2 Materials and methods

### 2.1 Study design and sampling

Grapevine (*Vitis rotundifolia*) varieties ‘Alachua’ and ‘Noble’ under open field cultivation (3 × 2.5 m) at the Lishui Plant Sciences Experiment Station (Nanjing, Jiangsu, China) were used. The study was conducted in 2022. The highest and lowest temperatures were 40 °C in August and -7 °C in December, and the rainfall level was 1,024.8 mm. The soil properties of the field are presented in **Supplementary Table S1**. Soil physicochemical properties (total nitrogen, available nitrogen, total phosphorus, available phosphorus, total potassium, available potassium) were determined according to a previous study (Bao, 2000). Samples from the four compartments rhizosphere, rhizoplane, root, and leaf were used for microbiological analysis. Furthermore, grapevine leaves were collected for transcriptomic and metabolic analyses. Three replicates were analyzed for each compartment in the present study. For each replicate, we included two grapevines. Three underground samples were collected from each grapevine, and six underground samples from two grapevines were mixed as one replicate. In addition, six leaves (leaf blade) were collected from each grapevine, while twelve leaves from two grapevines were mixed as one replicate. Underground samples were divided into rhizosphere, rhizoplane, and root endosphere compartments and subjected to the methods described by Edwards et al. (2015). In brief, the adhered bulk soil was removed from the root, the root was shaken with 50 mL sterile phosphate-buffered saline (PBS) solution (P1020, Solabio Science and Technology (Bei Jing) Co., Ltd, Beijing, China), and the soil was separated from fibrous root; this part of the soil was sampled as the rhizosphere compartment (RH). The root was sonicated for 30 s at 50 Hz with 15 mL sterile PBS solution, and the liquid PBS was used as the rhizoplane compartment (RP). The sonication procedure was repeated, and the root was used as the root endosphere compartment (RE). The leaf surface was sterilized using 75% ethanol (Hushi, Sinopharm Chemical Reagent Co., Ltd, Shanghai, China) (30 s), sterile ddH_2_O (five times), 0.1% mercuric chloride (Hushi, Sinopharm Chemical Reagent Co., Shanghai, Ltd, China) (5 min), and sterile ddH_2_O (five times). And then, the leaf was dried with sterile filter paper, and frozen in liquid nitrogen stored at -80 °C for leaf endosphere compartment (LE) DNA extraction.

### 2.2 Sample DNA extraction, amplicon sequencing (16S rRNA and ITS), and data processing and analysis

Four compartments (rhizosphere, rhizoplane, root, and leaf) DNA were extracted using the E.Z.N.A. ^®^ soil DNA Kit (Omega Bio-tek, Norcross, GA, USA), respectively. The DNA quality (purity, concentration, and integrity) was determined using a NanoDrop2000 (Nanodrop Technologies, Wilmington, DE, USA) and 1% agarose gel. Polymerase chain reaction (PCR) targeting the bacterial 16S rRNA V5–V7 region were performed using primer pairs 799F-1193R, the ITS1 region of fungal rRNA was amplified using the ITS1F_ITS2R primer pair (Jiao et al., 2019). Amplicons were purified (PCR Clean-Up Kit, Yuhua, China), quantified (Qubit 4.0, Thermo Fisher Scientific, USA). The library was constructed using NEXTFLEX Rapid DNA-Seq Kit (Bioo Scientific, USA). The purified amplicons were pooled at equimolar concentrations for paired-end sequenced (2–300 bp) on an Illumina platform (Miseq PE300, Illumina, San Diego, USA) according to the standard protocols by Majorbio Bio-Pharm Technology Co. Ltd. (Shanghai, China).

The raw sequences underwent quality filtering using fastp 0.19.6 and FLASH 1.2.7 (Oksanen et al., 2013; Shenhav et al., 2019). Discarding reads with quality < 20 over 50 bp or ambiguous bases, and assembling overlapping sequences (>10 bp, ≤ 0.2 mismatch). Samples were barcoded with dual-indexing (2-mismatch tolerance). OTU clustering at 97% identity (UPARSE v11) utilized Silva138 for bacteria and UNITE8 for fungi, followed by downstream analysis on Majorbio Cloud Platform (www.majorbio.com) (Ren et al., 2022).

To minimize the effects of sequencing depth on alpha and beta diversity measure, the number of 16S rRNA gene sequences from each sample were rarefied to 672053, which still yielded an average Good’s coverage of 98.85%. The number of ITS gene sequences from each sample were rarefied to 32153, which still yielded an average Good’s coverage of 99.96%. Alpha-diversity indices (Chao and Shannon) were calculated using the Mothur (v1.30.2). The Chao and Shannon were calculated to estimate microbial alpha-diversity. The similarity among the microbial communities was determined by principal-coordinate analysis (PCoA) using vegan package in R (v 3.3.1). Analysis of variance based on Turkey test was performed to determine the differences (*P* < 0.05) in bacterial and fungal alpha diversity indices and relative abundance of OTUs at the phylum and genus level among four grapevine compartments. Permutational multivariate analysis of variance (PERMANOVA) was performed using the vegan R package (Chen et al., 2018). Shared core microbes were identified according to the criteria established in a previous study (Jiao et al., 2019), with the following modifications: (i) abundant OTUs were > 0.01% and found in over 80% of all samples and (ii) OTUs from (i) were screened furthered for presence in over 50% of all leaf samples.

### 2.3 Leaf RNA extraction, high-throughput sequencing, and metabolic analysis

Part of the leaf samples described in section 2.1 was used for transcriptomic and metabolomic analysis. The RNAprep Pure Plant Kit (TIANGEN, China) was used to extract total leaf RNA. RNA degradation and contamination was monitored on 1% agarose gels. RNA purity was checked using the NanoPhotometer^®^ spectrophotometer (IMPLEN, CA, USA). RNA concentration was measured using a Qubit^®^ RNA Assay Kit and Qubit^®^2.0 Flurometer (Life Technologies, CA, USA). RNA integrity was assessed using the RNA Nano 6000 Assay Kit for the Bioanalyzer 2100 system (Agilent Technologies, CA, USA).

A total amount of 1 μg RNA per sample was used as input material for the RNA sample preparations. Sequencing libraries were generated using NEBNext^®^UltraTM RNA Library Prep Kit for Illumina^®^(NEB, USA) following manufacturer’s recommendations and index codes were added to attribute sequences to each sample. Then, PCR was performed with Phusion High-Fidelity DNA polymerase, Universal PCR primers, and Index (X) Primer. Finally, PCR products were purified (AMPure XP system) and library quality was assessed on an Agilent Bioanalyzer 2100 system. Sequencing was performed on the Illumina^®^ platform (NEB, USA) at Metware in Wuhan, Hubei, China. Raw data was filtered and qualitied using fastp (Chen et al., 2018). Then, clean reads were separately aligned to the reference genome *Vitis vinifera* 29760, with the orientation mode using HISAT2 (Kim et al., 2015). The fragments per kilobase of transcript per million (FPKM) mapped reads were calculated to evaluate gene expression, and computed using featureCounts v1.6.2/StringTie v1.3.4d (Burns et al., 2015; Gilbert et al., 2014).

Metabolic analysis was performed as described by Wang et al. (2019). Briefly, the leaf samples were freeze-dried and subjected to extraction using 70% methanol solution. After filtration, the extracts were analyzed using an ultra-performance liquid chromatography electrospray ionization mass spectrometry (UPLC-ESI-MS/MS) system (UPLC, ExionLC™ AD, https://sciex.com.cn/; MS, Applied Biosystems 6500 Q TRAP, https://sciex.com.cn/) (Dong et al., 2024). The mobile phase consisted of solvent A (pure water with 0.1% formic acid) and solvent B (acetonitrile with 0.1% formic acid) at a gradient of 95% A and 5% B; the gradient changed to 5% A and 95% B within 9 min, which was maintained for 1 min, and then 95% A and 5.0% B was adjusted within 1.1 min and maintained for 2.9 min. The flow velocity was set to 0.35 mL/min, and the column oven was set to 40°C. The injection volume was 4 μL. MS data were collected using Tandem mass spectrometry (MS/MS) (Applied Biosystems 4500 QTRAP, https://sciex.com.cn/) in positive and negative ion modes. The mass spectrometer parameters were optimized as follows (Dong et al., 2024): The source temperature was maintained at 550 °C, with electrospray ionization (ESI) configured in dual polarity mode (5500 V for positive ions, -4500 V for negative ions); ion source gas I, gas II, curtain gas were set at 50, 60, and 25 psi, respectively; and collision-activated dissociation was high. QQQ scans were acquired as MRM experiments with collision gas (nitrogen) set to medium. Qualitative and quantitative analysis of metabolites were performed using self-built Metware database (Metware Biotechnology Co., Ltd. Wuhan, China) (Dong et al., 2024). Briefly, the characteristic ions of each substance were screened out by the triple quadrupole rod, and the signal strength of the characteristic ions were obtained in the detector. The integration and correction of chromatographic peaks, and the relative content of the corresponding substances in the peak area of each chromatographic peak were calculated. To compare the contents of each metabolite in different samples, we calibrated the mass spectrum peaks detected by each metabolite in different samples based on the information of metabolite retention time and peak pattern (Fraga et al., 2010).

### 2.4 Integration of multi-omic analysis

First, the relative abundance of the top 10 bacterial and fungal genera was screened for further correlation analysis. Then, data on the stilbene compounds detected in the metabolomic analysis were filtered for the next correlation analysis. Correlations between the top 10 genera (bacteria and fungi, respectively) and stilbene compounds were evaluated using Spearman’s correlation coefficients on the Majorbio Cloud Platform (www.majorbio.com). Additionally, correlations between shared core microbes (bacteria and fungi, respectively) and stilbene compounds were also determined. Next, a weighted gene co-expression network analysis (WGCNA) of the top 500 RNAs according to expression level was performed to determine modules of highly correlated genes and identify gene modules related to stilbene compounds. WGCNA was performed using R software with the package WGCNA 1.69 (Wang et al., 2024a). Gene module networks were visualized using Cytoscape (version 3.8.2) Wang et al., 2024a).

## 3 Results

### 3.1 Effects of plant variety and compartment on the grapevinemicrobiome

**Figure 1:**
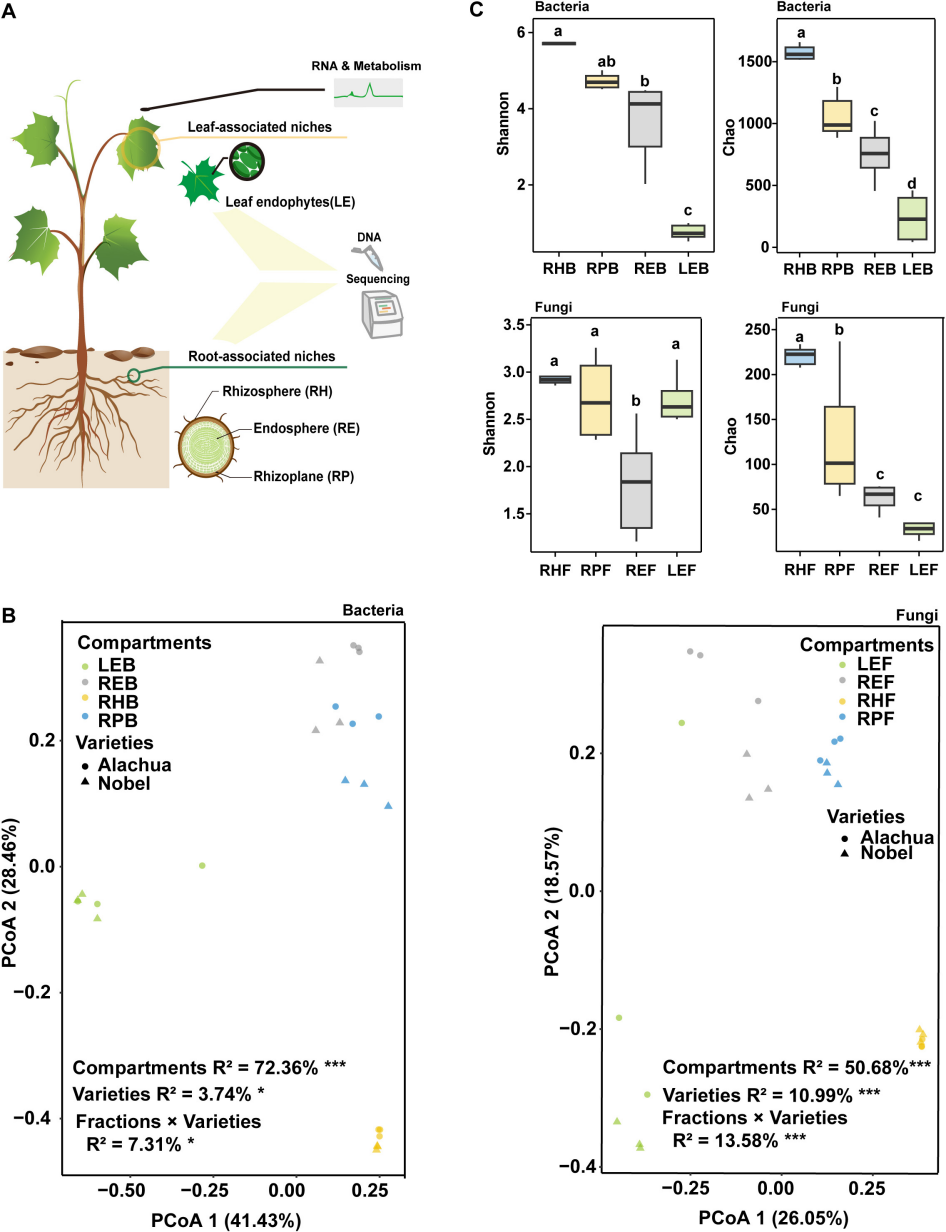
Effects of grapevine variety and compartments on grapevine microbiome. **(A)** Study design information. **(B)** Unconstrained principal coordinates analysis with Bray–Curtis distance across the entire dataset. (*p < 0.05, **p < 0.01, ***p < 0.001, PerMANOVA by Adonis). LEB, REB, RPB, and RHB indicate operational taxonomic units (OTUs) at the bacteria level from the leaf endosphere, root endosphere, rhizoplane, and rhizosphere, respectively. LEF, REF, RPF, and RHF indicate the OTUs at the fungal level from the leaf endosphere, root endosphere, rhizoplane, and rhizosphere, respectively. **(C)** Boxplot showing the distribution of Shannon and Chao indices for bacterial and fungal communities. Different letter indicates significant difference (Turkey test, *p* < 0.05).

As shown in **Figure 1B**, PCoA showed that the grapevine compartments were the primary drivers of grapevine bacterial and fungal compositions (bacteria: compartments R^2^ = 72.36%, *p* < 0.001; varieties R^2^ = 3.74%, *p* < 0.001; fungi: compartments R^2^ = 50.68%, *p* < 0.05; varieties R^2^ = 10.99%, *p* < 0.05). In addition, the bacterial and fungal beta-diversity analyses showed no significant differences (*p* > 0.05) in the same compartment between the two varieties (**Supplementary Figures S1**, **S2**). The bacterial alpha diversity results showed significant differences among the four compartments, with a decreasing trend from the rhizosphere to the leaf (**Figure 1C**). The fungal alpha diversity exhibited a different trend. The Chao index of the fungal community showed a decreasing trend from rhizosphere to root. The Shannon index showed no significance between the root and leaf endosphere (**Figure 1C**).

### 3.2 Changes in composition of grapevine-associated microbiomes along rhizosphere-leaf

**Figure 2:**
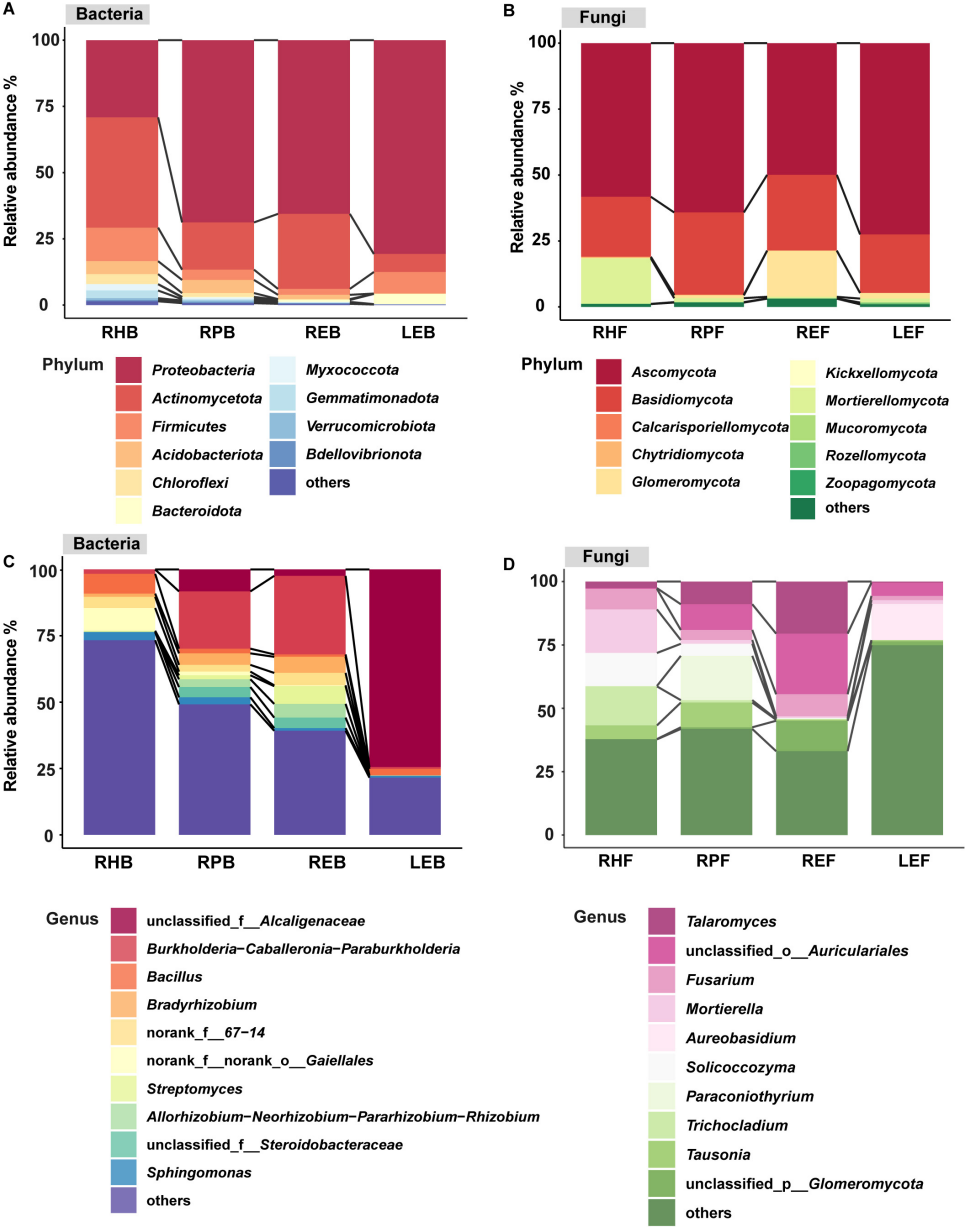
Composition of microbial community at bacterial and fungal levels. Phylum-level distribution of bacteria **(A)** and fungi **(B)** in different grapevine compartments. Genus-level distribution of bacteria **(C)** and fungi **(D)** in different grapevine compartments. LEB, REB, RPB, and RHB indicate samples from the leaf endosphere, root endosphere, rhizoplane, and rhizosphere at the bacteria level, respectively. LEF, REF, RPF, and RHF indicate samples from the leaf endosphere, root endosphere, rhizoplane, and rhizosphere at the fungal level respectively.

The bacterial and fungal community compositions in the four compartments were investigated. At the bacterial level, the grapevine bacterial microbiomes from the rhizosphere to the leaf were dominated by *Actinomycetota* and *Proteobacteria* at the phylum level, accounting for 6.79–80.74% (**Figure 2A**; **Supplementary Table S2**). *Proteobacteria* was the predominant phylum in the RH (29.14%), RP (68.81%), RE (65.58%), and LE (80.74%). *Chloroflexi* was more abundant in RH and RP than that in RE and LE, and its abundance significantly decreased from rhizosphere to leaf. (**Figure 2A**; **Supplementary Table S2**). *Actinomycetota*, *Acidobacteriota* and *Bdellovibrionota* were significant higher in root-related compartments than those in the leaf endosphere (**Figure 2A**; **Supplementary Table S2**). For the fungi, *Ascomycota* and *Basidiomycota* accounted for 22.22–75.52% of the phyla in the four compartments. *Chytridiomycota* and *Zoopagomycota* were only detected in root-related compartments (**Figure 2B**; **Supplementary Table S2**). *Calcarisporiellomycota*, *Kickxellomycota*, and *Mucoromycota* were not observed in the plant endosphere (**Figure 2B**; **Supplementary Table S2**). *Mortierellomycota*, *Chytridiomycota*, and *Zoopagomycota* were obviously higher in RH than in LE (**Figure 2B**; **Supplementary Table S2**).

The OTU distribution at the genus level of bacteria was also evaluated (**Figure 2C**; **Supplementary Table S3**). The relative abundances of norank_f:*67–14* was higher in root-related compartments than that in LE, and the relative abundances of norank_f:norank_o:*Gaiellales* and *Sphingomonas* significantly decreased from rhizosphere to leaf. The relative abundance of unclassified_f:*Alcaligenaceae* was more abundant in LE than that in root-related niches, while the relative abundance of *Bacillus* was more abundant in RH than that in other compartments (**Figure 2C**; **Supplementary Table S3**). For the fungi, the relative abundance of *Solicoccozyma* obviously decreased from RH to LE (**Figure 2D**; **Supplementary Table S3**). The relative abundance of *Aureobasidium* was significantly higher in leaf endosphere than that in root-associated compartments while the relative abundance of *Fusarium* was higher in root-associated compartments than that in leaf compartment (**Figure 2D**; **Supplementary Table S3**). The relative abundance of *Trichocladium* was higher in RH than that in other compartments (**Figure 2D**; **Supplementary Table S3**). The relative abundance of *Tausonia* was more abundant in root-related compartments than that in plant endosphere (**Figure 2D**; **Supplementary Table S3**).

**Figure 3:**
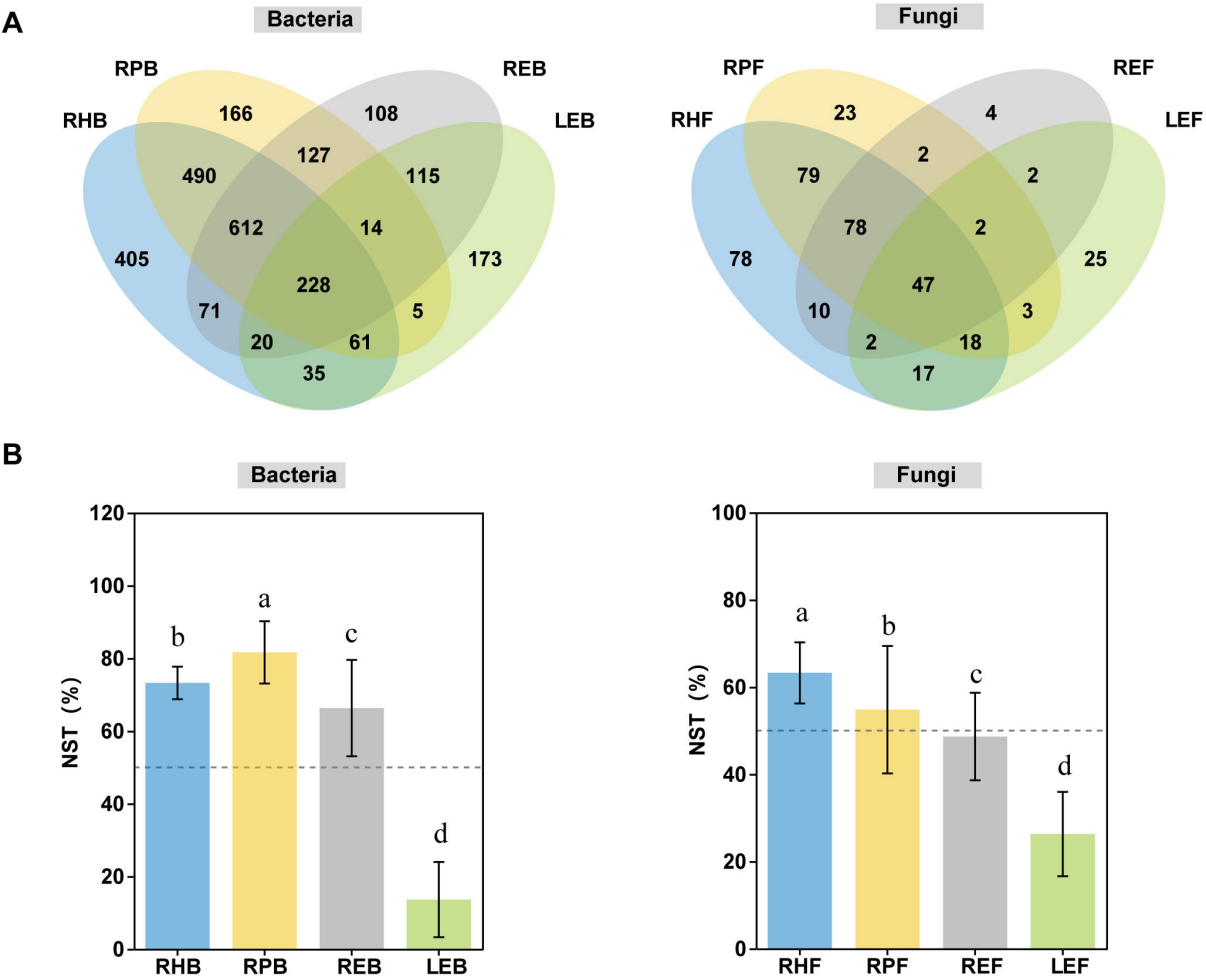
OTU-distribution characteristics from rhizosphere to leaf. **(A)** Venn diagram of overlapping OTUs enriched in different compartments. **(B)** Comparison of normalized stochasticity ratio (NST) among four different compartments at the bacterial and fungal levels.

Subsequently, we used a Venn diagram to evaluate the distribution of overlapping OTUs (**Figure 3A**). The results showed that 228 (8.67%) and 47 (12.05%) bacterial and fungal OTUs, respectively, were common to all compartments. The specific bacterial OTUs presented in only one compartment were 405 (15.40%), 166 (6.31%), 108 (4.11%), and 173 (6.58%) in the RH, RP, RE, and LE, respectively, and those of fungi were 78 (20%), 23 (5.9%), 4 (1.03%), and 25 (6.41%) OTUs in the RH, RP, RE, and LE, respectively. Additionally, a normalized stochastic ratio (NST) was used to quantify the role of deterministic and stochastic processes among the different compartments (**Figure 3B**). The NST values of the bacterial and fungal communities in the root-related compartments were significantly higher than those in the leaf endosphere (*p <* 0.001). The NST value exceeded the 50% threshold for bacterial communities in the RH, RP, and RE, implying that stochastic processes dominated the bacterial community assembly, and deterministic processes dominated the LE (13.61%). In the fungal community, stochastic processes dominated the RH and RP, and deterministic processes dominated the RE and LE (**Figure 3B**).

### 3.3 Correlation among leaf endosphere core microbiome, leaf metabolites, and leaf transcriptome

**Figure 4:**
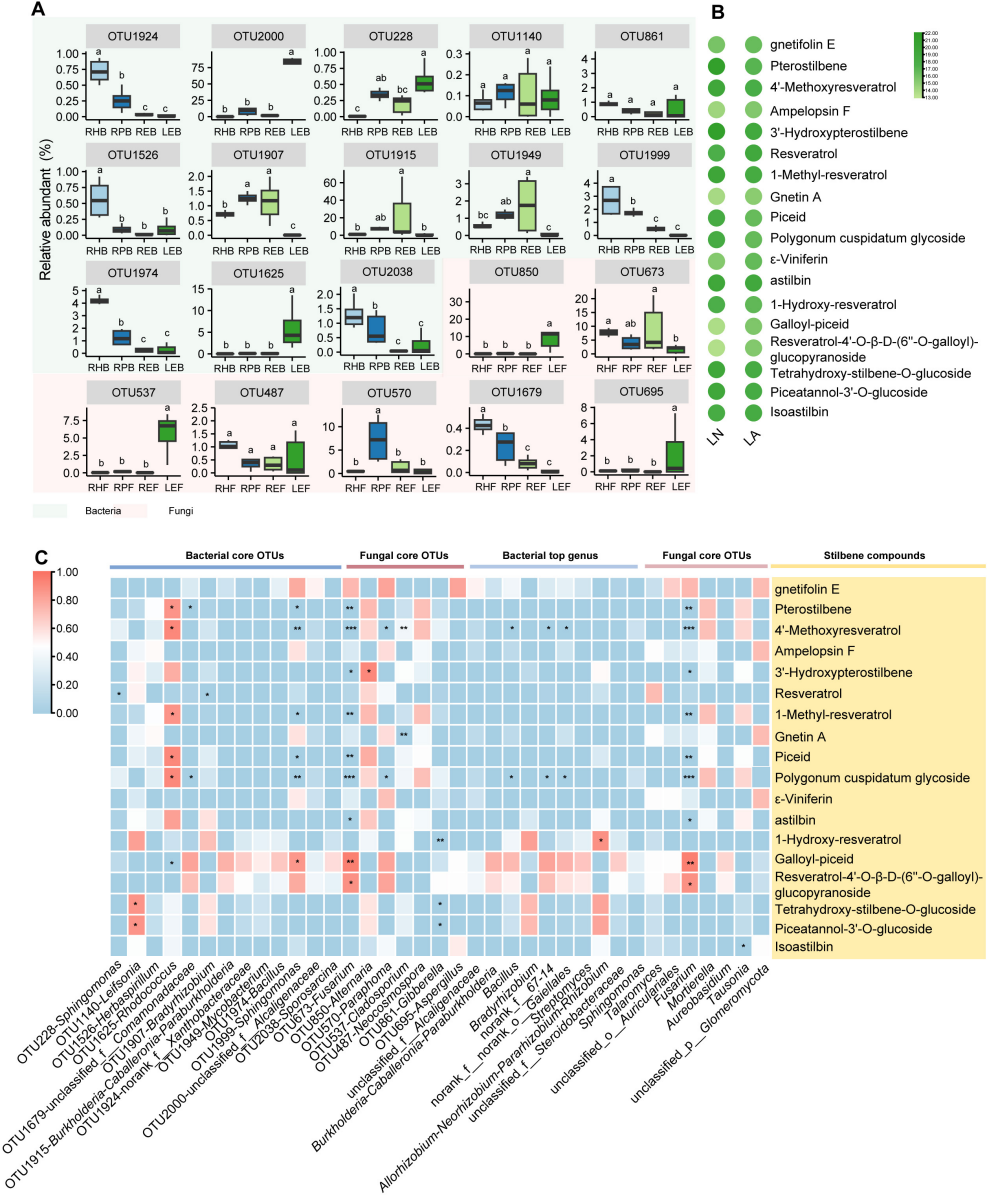
Correlation between microbes and stilbene compounds. **(A)** Relative abundance of shared core OTUs. **(B)** Heatmap of stilbene compounds content in leaf. Color indicates log_2_(stilbene content). **(C)** Correlation between microbial top 10 genera and stilbene compounds, shared core OTUs, and stilbene compounds at bacterial and fungal levels, respectively. Colors represent Spearman correlations. *p < 0.05, **0.001< *p <* 0.01, and ***p < 0.001.

To evaluate the association between stilbene metabolites and microbes, first we identify the shared core microbiota across the roots and leaves. Thirteen and seven OTUs were identified in the bacterial and fungal communities, respectively (**Supplementary Table S4**). The shared core OTUs were primarily classified as *Proteobacteria* (Bacteria), *Actinobacteria* (Bacteria), *Firmicutes* (Bacteria), and *Ascomycota* (Fungi) (**Supplementary Table S4**). For the bacterial level, the relative abundance of OTU1924 (*Proteobacteria*), OTU1526 (*Proteobacteria*), OTU1999 (*Proteobacteria*) and OTU1974 (*Firmicutes*) were more abundant in RH than those in other compartments, while the relative abundance of OTU1625 (*Actinomycetota*) and OTU2000 (*Proteobacteria*) were more abundant in LE than those in the other compartments (**Figure 4A**; **Supplementary Table S4**). Forfungal level, compared with the other compartments, OTU850 (*Ascomycota*), OTU537 (*Ascomycota*), and OTU695 (*Ascomycota*) were more abundant in LE, while OTU1679 (*Ascomycota*) was more abundant in RH and RP (**Figure 4A**; **Supplementary Table S4**).

In the metabolites analysis, a total of 18 stilbene compounds were detected (**Figure 4B**; **Supplementary Table S5**). We then conducted a Spearman correlation test in the four groups using 18 stilbene compounds with the top 10 bacterial genera, shared core bacterial OTUs, 10 fungal genera, and shared core fungal OTUs (**Supplementary Table S6**). As shown in **Figure 4C**, among bacterial core OTUs, OTU1625 (*Rhodococcus*) and OTU1999 (*Sphingomonas*) were clearly correlated with various stilbene compounds (pterostilbene, 4’-methoxyresveratrol, 1-methyl-resveratrol, piceid, polygonum cuspidatum glycoside, resveratrol-4’-O-β-D-(6’’-O-galloyl)-glucopyranoside). Among the fungal core OTUs, OTU1673 (*Fusarium*) was significant correlated with the stilbene compounds listed above and significantly correlated with 3’-hydroxypterostilbene and astilbin. OTU861 (*Gibberella*) was negative correlated with 1-hydroxy-resveratrol, tetrahydroxy-stilbene-O-glucoside, and piceatannol-3’-O-glucoside. Among the top 10 bacterial genera, *Bacillus*, norank_f:*67–14* and norank_f:norank_o:*Gaiellales* were negatively correlated with 4’-methoxyresveratrol and polygonum cuspidatum glycoside. *Allorhizobium*-*Neorhizobium*-*Pararhizobium*-*Rhizobium* was positively correlated with 1-hydroxy-resveratrol. Among the top 10 fungal genera, *Fusarium* was positively correlated with galloyl-piceid and resveratrol-4’-O-β-D-(6’’-O-galloyl)-glucopyranoside (**Figure 4C**).

**Figure 5:**
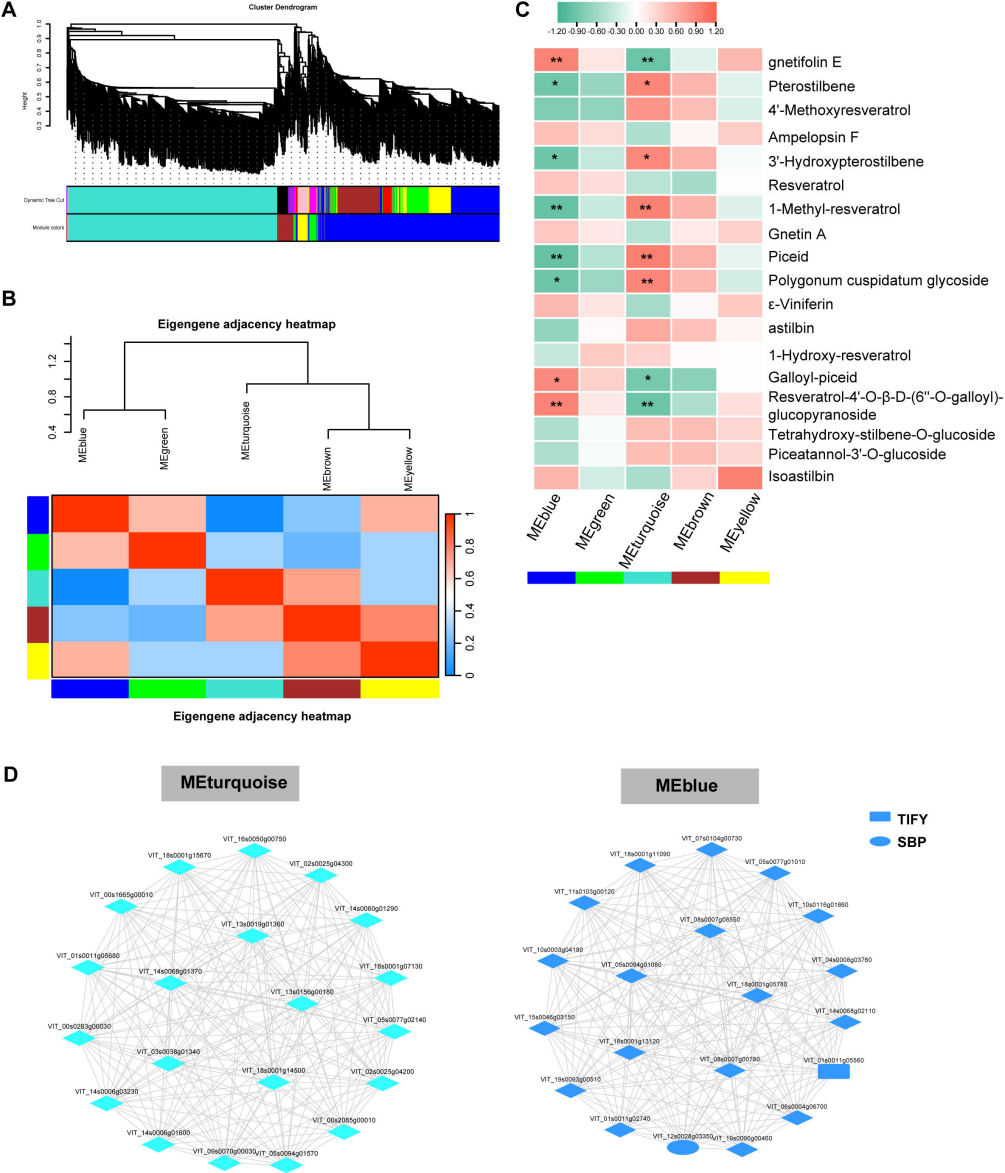
Weighted correlation network analysis modules of top 500 RNAs in leaf. **(A)** Tree graphs of 500 genes by hierarchical clustering of topological dissimilarities. **(B)** Correlation of different modules. **(C)** Heatmap showing modules–stilbene compound correlations. Colors represent Spearman correlations. Red indicates a positive correlation, and blue indicates a negative correlation. *P < 0.05, **0.001<*P < 0*.01, and ***P < 0.001. **(D)** Co-expression gene network of blue and turquoise modules.

The crucial plant genes involved in the regulation of stilbene compounds were identified. Subsequently, the top 500 genes were subjected to WGCNA (**Supplementary Table S7**). Given the limited sample size, the following Weighted Gene Co-expression Network Analysis (WGCNA) was presented as an exploratory, hypothesis-generating approach to identify potential key genes and pathways for future validation in larger cohorts. Five co-expression modules were identified (**Figure 5A**), and the results showed that the blue and green modules clustered into one group and the turquoise, brown, and yellow modules clustered into one group (**Figure 5B**). As shown in **Figure 5C**, the blue and turquoise modules had a significant content of various stilbene compounds (gnetifolin E, pterostilbene, 3’-hydroxypterostilbene, piceid, polygonum cuspidatum glycoside, galloyl-piceid, and resveratrol-4’-O-β-D-(6’’-O-galloyl)-glucopyranoside). Moreover, the blue and turquoise modules showed opposite correlations with the same stilbene compounds. The networks of the blue and turquoise modules contained 20 hub genes each (**Supplementary Table S8**; **Figure 5D**). In the co-expression network of blue module, two transcription factors (VIT_01s0011g05560, Tify; VIT_12s0028g03350, SBP) were identified.

## 4 Discussion

### 4.1 Plant compartments affect plant microbiome diversity and composition

This study revealed that the plant compartment and genotype and their interactions could affect grapevine bacterial and fungal assembly. However, when considering the alpha- and beta diversities, plant microbiomes were more sensitive to plant compartments than to plant genotypes (**Figures 1B, C**; **Supplementary Figures S1**-**S3**). This result is consistent with existing observations that plant compartments are the major contributors to microbiome variation compared with plant genotype and soil conditions (Brown et al., 2020; Xiong et al., 2021; Cregger et al., 2018). The variation in the plant continuum may be caused by the filter capacity of the plant (Santoyo, 2022; Lareen et al., 2016), especially the selection of root exudates, which can act as chemoattractants or carbon source that drive the microbial shift between the outside and inside of the root (Sasse et al., 2018). Furthermore, bacterial diversity, richness, and fungal richness showed a decreasing trend from the root to the leaf, and the variation in fungal diversity showed no significant gradient changes in different compartments (**Figure 1C**). These results are similar to existing observations in rice and *Populus* (Edwards et al., 2015; Cregger et al., 2018).

The alpha and beta diversities did not significantly differ between the two grapevine varieties in the same compartment (**Supplementary Figures S1**-**S3**). However, studies have demonstrated that plant genotypes also cause variations in microbial assembly. For example, Cregger et al. (2018) found that fungal diversity differed among tree genotypes while bacterial diversity did not. Studies have also indicated that the filtering capacity of microbes in the plant continuum is genotype-dependent (Brown et al., 2020; Bulgarelli et al., 2013). Some plant genotypes with weak filtering capacity may cause microbial similarity between the endosphere and rhizosphere in some *Medicago truncatula* (Brown et al., 2020). Our results were inconsistent with those in the literature, possibly because we analyzed only two varieties.

The major bacterial phyla observed in this study were largely Proteobacteria and Actinomycetota, and the primary fungal phyla were Ascomycota and Basidiomycota, which was similar to the findings of previous studies (Biget et al., 2024; Darriaut et al., 2022; Martínez-Diz et al., 2019). As reported, *Bacillus*, *norank_f:67-14*, *norank_f:norank_o:Gaiellales*, *Sphingomonas* (Chen et al., 2020a; Sun et al., 2023; Liu et al., 2024), *Fusarium* and *Mortierella* (Roncero et al., 2003; Li et al., 2020) were more abundant in root-associated compartments, while *unclassified_f:Alcaligenaceae* (Mastan et al., 2020; Acar et al., 2022), *Aureobasidium* (Boutin et al., 2024) were more abundant in the leaf endosphere. These results provide useful information for selecting sample compartments of culture strains.

### 4.2 Microbial community assembly process in plant compartments

Providing a better understanding of microbial community assembly will advance our comprehension of biodiversity maintenance mechanisms and ecosystem functioning processes (Li et al., 2022; Widder et al., 2016). The neutral community model is one of the commonly used theories to explain the assembly of microbial communities (Chave, 2004). However, the leaf endosphere community data did not fit the neutral model appropriately in the present study. Here, we evaluate microbial community assembly based on normalized stochasticity ratio (NST), which is consistent with other studies (Ning et al., 2019; Wang et al., 2023). As reported, deterministic and stochastic processes significantly influence the microbial community assembly (Wang et al., 2017; Martiny et al., 2011). In this study, the limited dispersal of bacterial and fungal taxa increased from the outside to the inside of the plant. Our data suggest a shift in the dominant assembly processes along the plant continuum: stochastic processes appear to be more influential in structuring rhizosphere and rhizoplane communities, whereas deterministic selection appears to dominate in the leaf compartment. This interpretation is advanced cautiously, acknowledging the limited sample size. This observation was similar to that of existing studies showing that stochastic processes were dominant in soil or rhizosphere community assembly while deterministic processes were dominant in the root compartment (Liu et al., 2023a; Zhuang et al., 2020). However, the community assemblies of the root endosphere bacteria and fungi presented the opposite results (bacteria, stochastic process; fungi, deterministic process). Previous studies have shown that distinct patterns of microbial host preference across root-associated and foliar environments. Fungal assemblages exhibit significantly stronger host-specific colonization patterns compared to bacterial populations (Li et al., 2022). This may be the reason for the differences in the bacteria and fungi communities between the root endospheres.

Stochastic processes dominated bacterial community assembly. For fungi, the dominant process shifted from stochastic in the rhizosphere and rhizoplane to deterministic in the root and leaf endosphere. The dominant role of deterministic processes in endosphere communities can be explained by host selection for bacteria (Biget et al., 2024). However, the opposite was observed by Zhong et al. (2022). The differences among these studies might have been caused by the differences in crops and sampling depths, as suggested by Benucci et al. (2023). Additionally, the neutral community model was not used in this study. Although the model is commonly used in the study of microbial community assembly (Zhong et al., 2022), the leaf endosphere community data did not fit the neutral model appropriately in the present study.

### 4.3 Association between plant metabolites and endophyte

Core microbial taxa are frequently observed across all or most individuals of the host (Risely, 2020). The significance of the core microbiome in stress reduction and growth promotion has been reported. For example, Luo et al. (2022) reported that the plant core rhizosphere microbiota contributed to host fitness and performance in metal-disturbed soils, and Hong et al. (2023) found that a unique core rhizosphere microbiome was associated with reduced banana *Fusarium* wilt disease. Another research showed that the core microbiomes of the rhizosphere and phyllosphere are associated with antibiotic resistance (Cernava et al., 2019).

The correlation between various stilbene compounds and shared core microbiome were conducted in the present study. Piceatannol-3’-O-glucoside and tetrahydroxy stilbene glucoside protected against aging hematopoietic stem cells, neuroinflammation and cancer, the role of them in plant has not been reported (Gao et al., 2023, 2025; Wang et al., 2024b). These two compounds were positively correlated with *Leifsonia* (**Figure 4C**). As an endophytic bacterial strain of stem and leaf, *Leifsonia* sp. ku-ls could mitigate plant oxidative stress caused by stress conditions. However, the mechanism of *Leifsonia* in mitigating oxidative stress has not been clarified (Battu and Ulaganathan, 2020). Resveratrol-4’-O-β-D-(6’’-O-galloyl)-glucopyranosid and galloyl-piceid showed positive correlation with *Fusarium* (**Figure 4C**). While the relative abundance of *Fusarium* and pterostilbene, 4’-methoxyresveratrol, 3’-hydroxypterostilbene, 1-methyl-resveratrol, piceid, polygonum cuspidatum glycoside, astilbin showed obviously negative correlation, respectively (**Figure 4C**). The total stilbene content in the *V. amurensis* cell suspension significantly increases after culture cocultivation with *Fusarium* sp (Aleynova et al., 2021). However, the stilbene compound categories detected in previous studies and the present study differed. Various *Fusarium* species are important pathogens to crops worldwide (Stępień, 2020; Ekwomadu and Mwanza, 2023). Nonetheless, the results indicated that resveratrol-4’-O-β-D-(6’’-O-galloyl)-glucopyranosid and galloyl-piceid may play key roles in the biocontrol of *Fusarium* species. Unexpectedly, *Fusarium* was negatively correlated with various stilbene compounds. The correlation of *Sphingomonas* and stilbene compounds showed a similar trend with that of *Fusarium* and stilbene compounds (**Figure 4C**). *Sphingomonas* has various roles in plants and can be enriched in disease-resistant rice seeds (Matsumoto et al., 2021) and cause bacterial dry rot of mango (Liu et al., 2018). In contrast, *Rhodococcus* is positively correlated with these stilbene compounds (**Figure 4C**). *Rhodococcus* is known for its strong resistance to abiotic stress and has been widely explored (Wang et al., 2020; Kuhl et al., 2021). However, the relation between the strong resistance of *Rhodococcus* and stilbene compounds remains to be explored. Another interesting finding was that *Alternaria* was positively correlated with 3’-hydroxypterostilbene (**Figure 4C**). An endophytic fungus (*Alternaria* sp. MG1) isolated from grape could produce resveratrol (Shi et al., 2012). In total, significant correlations were observed between stilbene levels and multiple microbial taxa. Although these relationships do not establish causality, the pattern is suggestive: contrary to initial expectations, negative associations predominated, with only a few taxa showing positive correlations.

Furthermore, we used WGCNA to identify key genes that may participate in the regulation of stilbene compounds. In the turquoise module, several hub genes were significantly correlated with stilbene compounds. However, the mechanisms of these genes in regulating stilbene compounds have not been reported. In the blue module, we also found several hub genes, including two transcript factors (TIFY and SBP), that were significantly correlated with stilbene compounds. Similarly, no evidence has been found that these genes were correlated with stilbene. Previous studies showed that SBP of grape participates in enhancing the tolerance to abiotic stress and floral transition (Hou et al., 2018). Moreover, TIFY has been found to increase plant tolerance to disease (Yu et al., 2019). The increasing tolerance to stress may be related to stilbene compounds. A key limitation of this study is the small cohort size, which precludes a statistically robust WGCNA. While the analysis identified candidate hub genes and modules, these findings must be considered preliminary and require confirmation in independent, larger-scale studies.

## 5 Conclusion

This study systematically investigates the interconnected grapevine microbiome from root to leaf. We identify a conserved, cross-compartment core microbiota and establish significant associations between these core microbial members and key host-derived stilbene compounds. Notably, host genes related to stilbene biosynthesis were linked to this core microbiome, suggesting a coordinated gene-metabolite-microbe network. These findings advance our understanding of plant-level microbiome organization and highlight the potential of core microbes as functional mediators of host metabolism. Future work should focus on experimentally validating the causal roles of these core taxa, potentially informing microbiome-based strategies to enhance grapevine health and secondary metabolism.

## Data Availability

The datasets presented in this study can be found in online repositories. The names of the repository/repositories and accession number(s) can be found below: https://www.ncbi.nlm.nih.gov/, PRJNA1195708. https://www.ncbi.nlm.nih.gov/, PRJNA1196027.

## Supplementary material

Supplementary Figure 1 Bacterial community structure beta-diversity difference analysis in different grapevines. RHNB, Rhizosphere bacteria in Nobel; RHAB, rhizosphere bacteria in Alachua; RPNB, Rhizoplane bacteria in Nobel; RPAB, Rhizoplane bacteria in Alachua; RENB, Root bacteria in Nobel; REAB, Root bacteria in Alachua; LENB, Leaf bacteria in Nobel; LEAB, Leaf bacteria in Alachua.

Supplementary Figure 2 Fungal community structure beta-diversity difference analysis in different grapevines. RHNF, Rhizosphere fungi in Nobel; RHAF, rhizosphere fungi in Alachua; RPNF, Rhizoplane fungi in Nobel; RPAF, Rhizoplane fungi in Alachua; RENF, Root fungi in Nobel; REAF, Root fungi in Alachua; LENF, Leaf fungi in Nobel; LEAF, Leaf fungi in Alachua.

Supplementary Figure 3 Shannon’s and Chao’s indices of bacterial and fungal communities in different grapevines.

Supplementary Table 1 Soil physicochemical properties.

Supplementary Table 2 Relative abundance in grapevine compartments at phylum level.

Supplementary Table 3 Relative abundance in grapevine compartments at genus level.

Supplementary Table 4 Taxa of shared core operational taxonomic units (OTUs) identified across four compartments.

Supplementary Table 5 Stilbene compounds detected in metabolites of Alachua and Nobel leaves.

Supplementary Table 6 Correlation between stilbene compounds and shared core microbes, and top 10 genus at fungal and bacterial level.

Supplementary Table 7 Top 500 expressed genes in Alachua and Nobel leaves.

Supplementary Table 8 Weight of module identified using weighted gene co-expression network analysis.
