# Supplementary figures and images for: Deciphering the profiles of grapevine microbiomes from rhizosphere-to-leaf compartments using multi-omic analysis
Source: Front Plant Sci. 2026 Jan 26;16:1734057. doi: 10.3389/fpls.2025.1734057 (PMC12883825; doi:10.3389/fpls.2025.1734057)

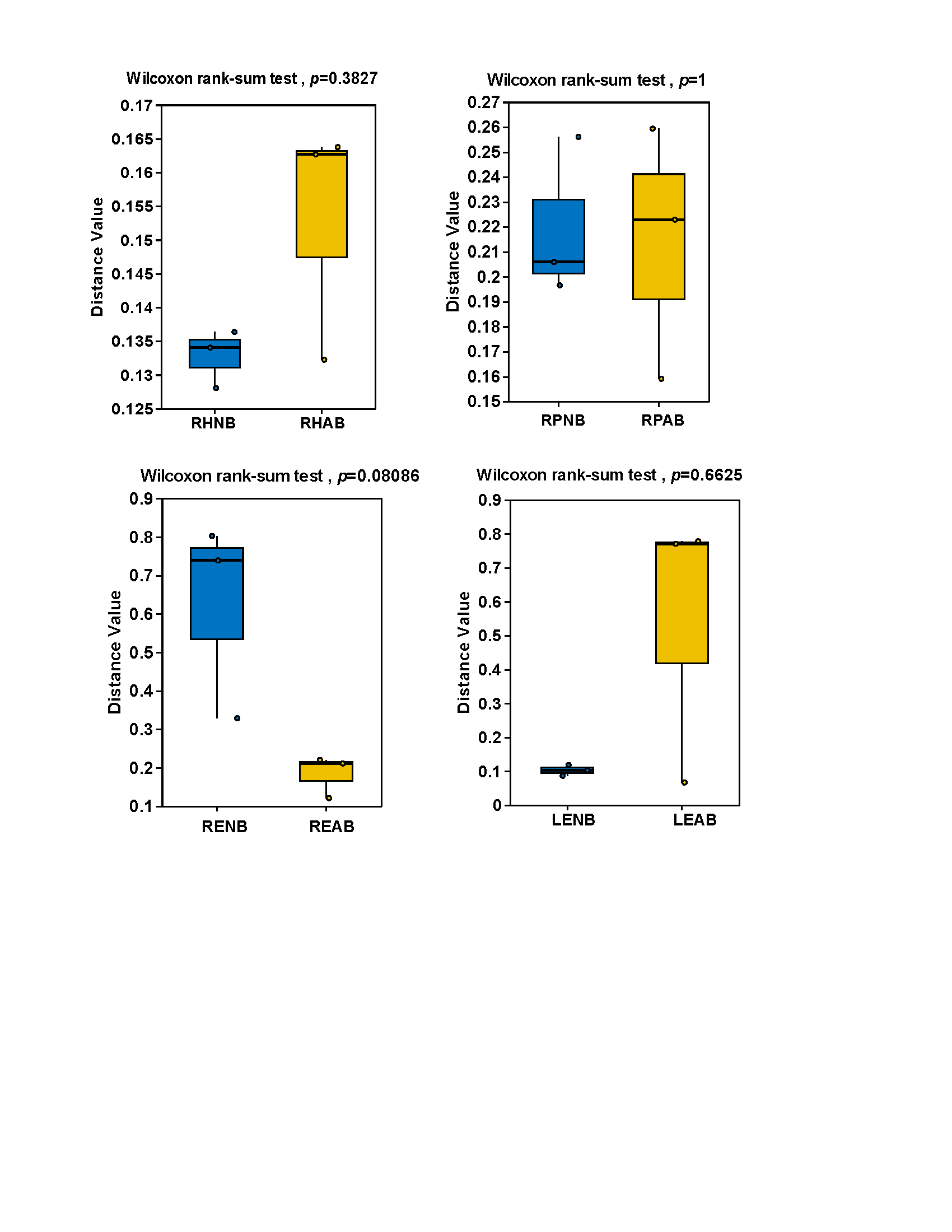

Supplement: Supplementary Figure 1 — Bacterial community structure beta-diversity difference analysis in different grapevines. RHNB, Rhizosphere bacteria in Nobel; RHAB, rhizosphere bacteria in Alachua; RPNB, Rhizoplane bacteria in Nobel; RPAB, Rhizoplane bacteria in Alachua; RENB, Root bacteria in Nobel; REAB, Root bacteria in Alachua; LENB, Leaf bacteria in Nobel; LEAB, Leaf bacteria in Alachua. [file Image1.tiff]

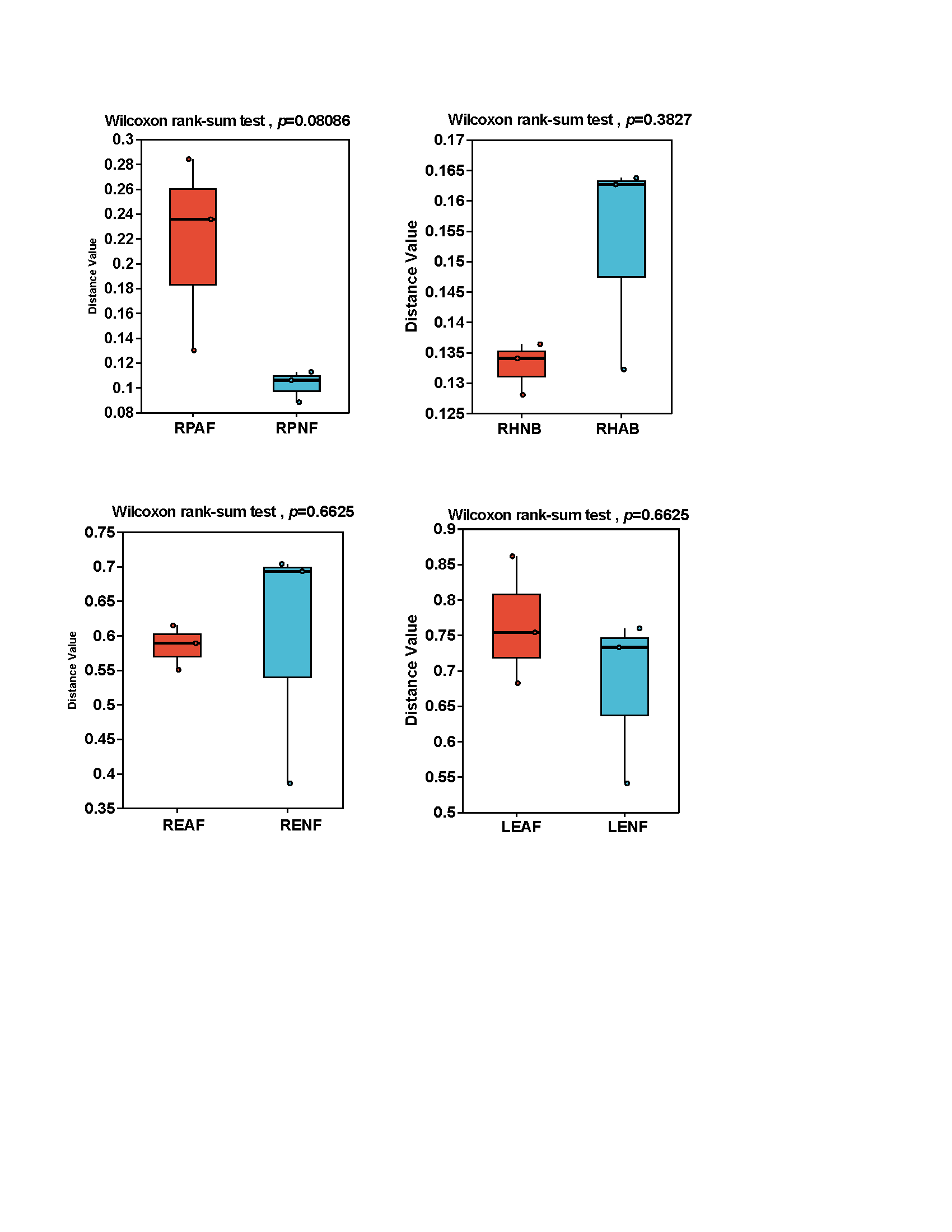

Supplement: Supplementary Figure 2 — Fungal community structure beta-diversity difference analysis in different grapevines. RHNF, Rhizosphere fungi in Nobel; RHAF, rhizosphere fungi in Alachua; RPNF, Rhizoplane fungi in Nobel; RPAF, Rhizoplane fungi in Alachua; RENF, Root fungi in Nobel; REAF, Root fungi in Alachua; LENF, Leaf fungi in Nobel; LEAF, Leaf fungi in Alachua. [file Image2.tiff]

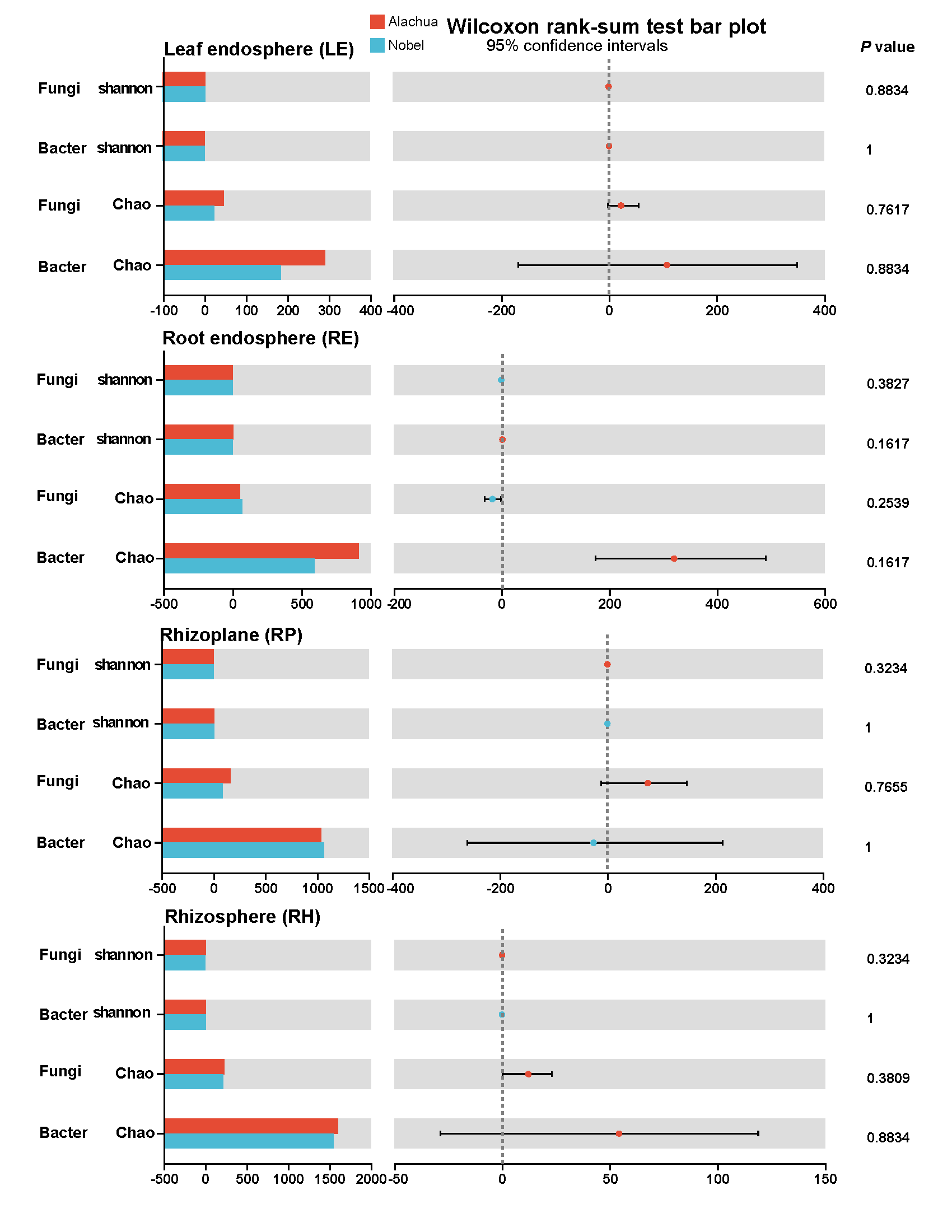

Supplement: Supplementary Figure 3 — Shannon’s and Chao’s indices of bacterial and fungal communities in different grapevines. [file Image3.tiff]
